# Two years of innovative dermatological care: the first public health consultation service for the transgender and gender diverse community in Argentina^☆^

**DOI:** 10.1016/j.abd.2024.03.003

**Published:** 2024-10-22

**Authors:** Lola Kuperman Wilder, Valeria Orsi, Gonzalo Chebi, Maria Agustina Balague, Luciana Cabral Campana

**Affiliations:** a Dermatology Department, Hospital Ramos Mejía, Buenos Aires, Argentina; b Institute of Calculus, Faculty of Exact and Natural Sciences, University of Buenos Aires, Buenos Aires, Argentina

**Keywords:** LGTBQ+ dermatology, Trans men, Trans woman, Inclusive dermatology

## Abstract

**Background:**

The LGBTQI + community encounters distinct healthcare challenges due to discrimination and inadequate understanding of their needs. Dermatologists play a crucial role in addressing this by fostering inclusiveness, recognizing individual concerns, and adopting tailored approaches, thereby promoting a more equitable healthcare system.

**Objective:**

To address the need for an inclusive healthcare space, the authors established the first dermatological practice exclusively for transgender and non-binary patients. This article presents a comprehensive two-year experience in a public hospital.

**Methods:**

The authors conducted a retrospective and descriptive study, analyzing the medical records of 114 patients evaluated at a specialized dermatological practice between June 2021 and May 2023. Key variables assessed included self-identified gender, age, residence, access to private healthcare, human immunodeficiency virus status, hormonal treatment, surgical interventions, consultation motives, employment stability, and family support during gender expression transition.

**Results:**

The present study included 114 patients, 49.1% trans men, 39.5% trans women, and 8.8% non-binary individuals. Trans men, on average younger than trans women (p < 0.001), predominantly sought care for body modification-related concerns, particularly acne and androgenetic alopecia. In contrast, trans women exhibited a more diverse range of consultation motives typically unrelated to hormonal or surgical procedures.

**Study limitations:**

This study is retrospective and limited in geographic scope. Additionally, the patient population lacked diversity in terms of Black ethnicity.

**Conclusions:**

The pioneering dermatological practice for transgender and non-binary patients exemplifies healthcare equity and cultural competence. Effective LGBTQI + healthcare requires addressing unique dermatological concerns while fostering inclusiveness and continuous learning within the medical community.

## Introduction

Dermatologic practices should prioritize inclusivity and impartiality, especially for transgender and gender-diverse patients. Dermatologists should be well-equipped to offer comprehensive and interdisciplinary care to individuals choosing hormonal and surgical interventions for bodily changes.^1^

Creating a safe space is imperative to provide high-quality healthcare to LGBTQI + individuals, particularly given the systematic discrimination they face within both society and the healthcare system. These disparities manifest in elevated rates of sexually transmitted infections, reduced participation in preventative cancer screenings, heightened prevalence of mental health conditions, adverse employment conditions, and diminished family support.^2^

In 2021, the authors took a proactive step by establishing the first cost-free dermatological practice exclusively dedicated to transgender and non-binary patients within a public hospital. This article presents a comprehensive account of the two-year experience working with this community.

## Methods

The authors conducted a retrospective and descriptive study encompassing all patients who sought evaluation at the transgender and non-binary dermatological practice within the Hospital José María Ramos Mejía between June 2021 and May 2023. This study analyzed the medical records of 114 patients, considering several key variables: self-identified gender, age, place of residence, access to private healthcare, Human Immunodeficiency Virus (HIV) status, duration and nature of hormonal treatment, engagement in surgical procedures, reasons for seeking consultation, employment status (including whether it was formal or informal), and the extent of family support during the gender expression transition. The statistical analysis was performed with R, the Exact Fisher Test was used to compare proportions, and the Two-sample Wilcoxon Test was used to compare the median of continuous variables. The authors used Logistic Regression to measure associations between continuous and binary variables.

## Results

A total of 114 patients were evaluated in this study (Table 1). The predominant gender identity among patients was trans men (TM), comprising 49.1% (n = 56), followed by trans women (TW) at 39.5% (n = 45), non-binary gender at 8.8% (n = 10), and others, including fluid gender identities.

**Table 1:** Demographic characteristics of the study population.

Variable	Overall Study Population	Trans Men (TM)	Trans Women (TW)
Total number of patients	114	56	45
Mean age (years)	29.6	26.9	33.4
City residence (%)	65.8	45.5	54.5
Access to private healthcare (%)	29.8	64.5	35.5
History of HIV (%)	15.2	0.0	38.6
Undergoing hormonal treatment (%)	63.2	85.7	52.3
Gender-affirming surgeries (%)	23.7	66.7	29.6
Most Frequent Reasons for Consultation	1- Acne (34.2%)	1- Acne (57.1%)	1- Acne (13.6%)
	2- Androgenic alopecia (9.6%)	2- Androgenic alopecia (12.5%)	2- Genital Warts (11.4%)
	3- Genital warts (6%)	3- Keloids (5.4%)	

Key findings in a 2-year experience of a public consultation service for the transgender and gender-diverse community (n = 114).

The average age of the patients was 29.6 years, with the youngest being a 12-year-old TM and the oldest a 60-year-old TW. TW were older than TM (p < 0.001), with average ages of 33.4 and 26.9 years, respectively.

The healthcare facility where this specialized practice was established is situated in the capital city of Buenos Aires. The majority of these patients, constituting 65.8% (n = 75), resided within the city, while 34.2% (n = 39) lived in areas outside the city.

In Argentina, where the healthcare system is a mixed model, a substantial portion of 70.1% (n = 80) of patients lacked access to private healthcare. Notably, those with private coverage (29.8% or n = 34) opted to utilize public hospital services for consultations.

A history of HIV was documented in 15.2% (n = 17) of the patients, and significantly, all of these cases were among TW. HIV-positive TW accounted for 38.6% of the total TW in the study (n = 17), with an average age of 39 years. Among these 17 TW, 41.1% (n = 7) were engaged in sex work. The prevalence of HIV infections was higher among TW compared to TM (p < 0.001).

In terms of hormonal therapy, 63.2% of the patients (n = 72) were undergoing treatment. A significant proportion of TM actively received testosterone treatment (either topically or intramuscularly), constituting 85.7% (n = 48) of this subgroup. Approximately half of the TW, 52.3% (n = 23), were undergoing hormonal treatment with estrogen, often in combination with spironolactone. Interestingly, within the non-binary gender category, nearly all patients abstained from hormonal treatment (90%, n = 9), with the exception of one individual undergoing estrogen and spironolactone therapy. Notably, TM were more frequently engaged in hormonal treatment than TW (p < 0.0001). On average, the duration of hormonal treatment was 28 months for TM and 20 months for TW.

A total of 27 patients (23.7%) underwent gender-affirming surgeries, with TM comprising 66.7% (n = 18) of this subgroup, followed by 29.6% (n = 8) TW and 3.7% (n = 1) non-binary individuals. Among TM, bilateral mastectomy was the predominant surgical procedure (32.1%; n = 18), while TW predominantly underwent mammoplasty (13.3%; n = 6), with additional cases involving phalloplasty and scrotoplasty (2.2%; n = 1, respectively).

Patients sought consultation for a variety of reasons. Acne, androgenetic alopecia, and genital warts were the most frequent concerns across the entire patient population, accounting for 34.2% (n = 39), 9.6% (n = 11), and 6% (n = 7) of patients.

**Fig. 1:**
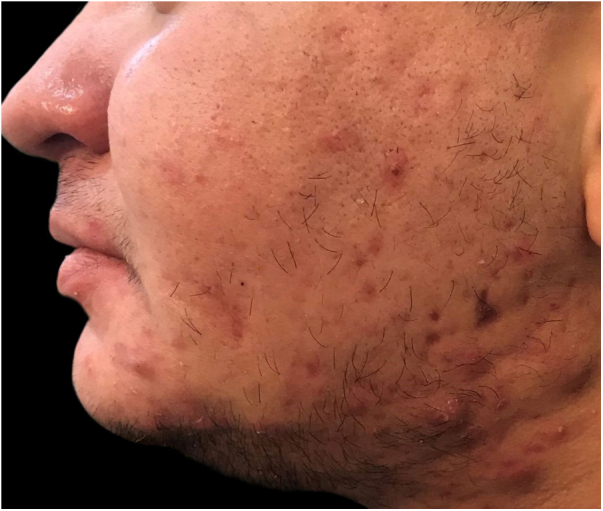
Nodular-cystic acne in a trans man developed three months following the initiation of intramuscular testosterone therapy.

**Fig. 2:**
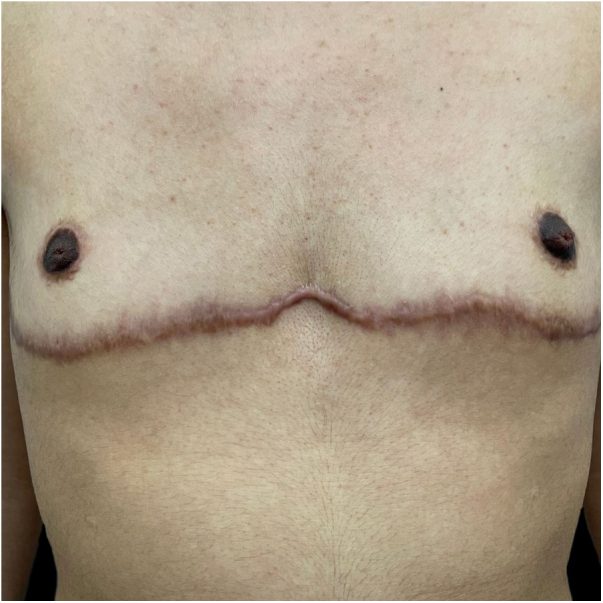
A trans man presented with keloid scars following bilateral mastectomy.

Interestingly, TM often sought medical attention for issues related to body modification, encompassing both surgical and hormonal aspects. Acne represented the primary motive for consultation in 57.1% (n = 32) (Fig. 1). Androgenetic alopecia emerged as the second most common reason for consultation affecting 12.5% (n = 7), followed by keloids in 5.4% (n = 3) (Fig. 2).

In the case of TW, there were multiple reasons for seeking consultation, regardless of whether they were on hormonal therapy or not. Acne and genital warts were the predominant concerns, affecting 13.6% (n = 6) and 11.4% (n = 5) of patients, respectively. These issues were followed by melasma, onychomycosis, siliconomas, atopic dermatitis, eczema, and nevus control.

As for non-binary individuals, their consultations encompassed a wide array of concerns, including androgenic alopecia, psoriasis and various fungal infections, among others.

Subsequent to the first year of establishing this specialized practice, it became evident that inquiries regarding patients' social and family backgrounds were crucial for comprehending this population. During inquiries about stable employment, the authors observed a parallel employment rate of 68% (n = 28) for TM and 65% (n = 34) for TW. However, it is noteworthy that a significant portion of TW (two-thirds) did not hold registered employment (28.8%; n = 13 of TW were sex workers), while nearly half of the TM did. Concerning family support, TM reported the highest level, with 58.1% (n = 21) indicating adequate support, followed by TW at 22.2% (n = 8).

## Discussion

The groundbreaking Gender Identity Law (26.743) was established in Argentina in 2012 and promotes that every person has the right to recognition, the free development of their personality and to be treated in accordance with their gender identity.^3^ Despite this law, transgender individuals still face various obstacles in accessing the healthcare system.

An analysis of international dermatological literature and the LGBTQI + community conducted between 1980 and 2020 revealed an increase in scientific publications starting from 2015. 62.2% of the articles are focused on topics such as HIV and infectious diseases, 20.4% on dermatological conditions, 12.4% on culturally competent medical care, and 4.9% on gender reassignment surgeries.^4^

To further promote diversity in continuing medical education, it is necessary to challenge the binary concept of male/female that currently underpins it. The transgender population includes people who do not identify with their assigned gender at birth, as well as those who identify with non-binary or queer gender expressions, which do not fit within the binary category (neither transgender men nor transgender women).^3^, ^5^, ^6^, ^7^

Transgender dermatology is an unexplored and fertile field. To our knowledge, this is the first retrospective study reviewing the experience of dermatological consultations in a practice created specifically for this population. Understanding the demographics and the main reasons for consultation in the transgender and gender-diverse community is the key to providing an inclusive and friendly healthcare system.^1^, ^8^

Also, understanding the background is essential to breaking down barriers in healthcare for the transgender community. Depression, discrimination, suicide attempts, eating disorders, abuse, lack of family and peer support, as well as stable housing from a young age, and limited access to private healthcare and social services are important factors to consider when caring for this population.^1^, ^2^, ^3^, ^9^ A notable 34.2% of these patients traveled from outside the city to access dermatological and inclusive care. This statistic underscores the significance of having healthcare practices of this nature.

Despite the increasing social and medical visibility of the LGBTQ + community, the main barrier they face is stigma. This community has a critical need for a safe and inclusive consultation space where they can be assessed and treated without prejudice.^2^

Jia et al. have formulated a series of recommendations for creating a friendly environment for the LGBTQ + community. Administrative, cleaning, and maintenance staff in healthcare institutions should be trained to interact with this population. During the interview, it is crucial to ask for the patient's chosen name and preferred pronouns and healthcare providers can state their own. Avoiding heteronormative assumptions is essential, as this can lead to patient discomfort and prevent the disclosure of crucial medical information.^10^

The primary reason for seeking medical consultation among these patients was acne, particularly prevalent in TM. In a comprehensive retrospective study encompassing 988 TM initiating testosterone therapy, it was observed that acne prevalence increased from 6.3% prior to treatment initiation to 31.1% after an average of 3.4 years of testosterone therapy. It is worth noting that the timing of hormone treatment initiation also played a significant role in the development of acne, with the youngest individuals showing a higher propensity for its onset. Similar to the present study, the diagnosis of acne was most frequently established within the initial 6 months of hormone therapy and exhibited a decreasing trend over the subsequent 2-year period.^11^

Androgenic alopecia was the second most frequent reason for consultation. The predominant treatment involved a combination of topical and oral minoxidil (5% and 2.5–5 mg, respectively) and a subset of patients underwent treatment with either topical or oral minoxidil. Finasteride was reserved for TM who had already developed secondary sexual characteristics, with careful consideration of pregnancy desires.

**Fig. 3:**
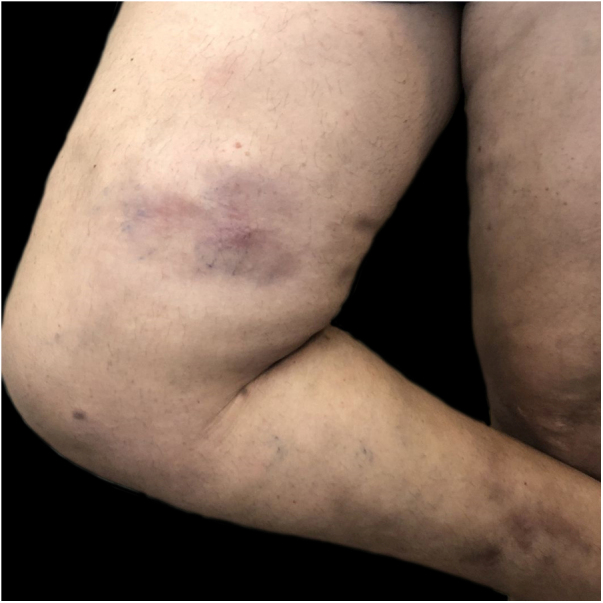
A trans woman with a history of silicone injections in the buttocks a decade ago presented with siliconomas on the inner side of her right thigh.

Regarding TW, the management of siliconomas posed significant challenges. The authors employed a therapeutic approach combining topical corticosteroids and oral minocycline (200 mg/day) in collaboration with plastic surgeons (Fig. 3). In one case, an ulcerated siliconoma led to the discovery of squamous cell carcinoma upon biopsy. Also, atopic dermatitis in a TW was exacerbated in the presence of estrogen therapy, although this was relatively rare. Implementing measures to improve skin barrier functions before commencing hormonal treatment proved pivotal in preventing exacerbations.

In accordance with a guide developed by the Argentine Ministry of Health to inform and educate healthcare workers, only 9% of TW held formal employment, with 15% in informal work. A staggering 70% of TW relied on sex work for economic survival. In contrast, TM fared considerably better, with 85% reporting employment. Nearly half had informal jobs, while one-third enjoyed formal employment and some received family support.^3^ The present study observed similar employment rates across both groups. Notably, TW had a higher proportion of unregistered work compared to TM, while also showing a lower percentage of sex workers than the Argentinian guide.

Dermatology, with its comprehensive expertise, is well-positioned to provide inclusive care for the transgender community, addressing various aspects of this population’s health. This emphasizes the imperative for a paradigm shift in dermatologic training and education, a transition towards a more inclusive approach with a dedicated effort to recognize and address the specific needs of the transgender community.^10^

## Conclusion

In conclusion, through a retrospective analysis of 114 patients in the two-year experience in the first dermatological practice in Argentina exclusively dedicated to transgender and non-binary patients, the authors have observed a diverse spectrum of dermatological concerns and differences between TW and TM.

Among TW, their reasons for seeking medical consultations are typically unrelated to hormonal or surgical procedures. Furthermore, this subgroup tends to be older, with approximately half of the sample currently utilizing hormonal therapy. It is imperative that during the medical interview, healthcare providers actively inquire about potential illicit silicone usage.

Conversely, TM consulted the practice for concerns primarily related to surgical or hormonal interventions, and dermatologists must stay informed about advancements in this field. This subgroup tends to be younger and exhibits a greater tendency to utilize hormonal therapy.

It is crucial to recognize the heterogeneity within this population and adopt an individualized, patient-centered approach. Furthermore, the study shed light on the broader social and economic aspects affecting transgender patients, including employment stability and family support. The present findings underscore the necessity for inclusive healthcare spaces and the importance of understanding the demographics and specific challenges faced by transgender individuals.

Despite the study's limitations, such as its retrospective nature and limited geographic scope, findings contribute to the growing body of knowledge in transgender dermatology. Further research on this population is essential to provide them with inclusive and quality care.

During the preparation of this work, the authors used Chat GPT in order to check and correct grammar. After using this tool/service, the authors reviewed and edited the content as needed and take full responsibility for the content of the publication.

## Financial support

None declared.

## Authors’ contributions

Lola Kuperman Wilder: The study concept and design; data collection, or analysis and interpretation of data; writing of the manuscript or critical review of important intellectual content; data collection, analysis and interpretation; effective participation in the research guidance; intellectual participation in the propaedeutic and/or therapeutic conduct of the studied cases; critical review of the literature; final approval of the final version of the manuscript.

Valeria Orsi: Data collection, analysis and interpretation of data; data collection, analysis and interpretation; intellectual participation in the propaedeutic and/or therapeutic conduct of the studied cases; final approval of the final version of the manuscript.

Gonzalo Chebi: Data collection, analysis and interpretation of data; statistical analysis; data collection, analysis and interpretation; intellectual participation in the propaedeutic and/or therapeutic conduct of the studied cases; final approval of the final version of the manuscript.

Maria Agustina Balague: Data collection, or analysis and interpretation of data; data collection, analysis and interpretation; final approval of the final version of the manuscript.

Luciana Cabral Campana: The study concept and design; writing of the manuscript or critical review of important intellectual content; effective participation in the research guidance; intellectual participation in the propaedeutic and/or therapeutic conduct of the studied cases; final approval of the final version of the manuscript.

## Conflicts of interest

None declared.

## References

[1] Sullivan P., Trinidad J., Hamann D. (2019). Issues in transgender dermatology: a systematic review of the literature. J Am Acad Dermatol..

[2] Bonati L.M., Jagdeo J. (2020). A new era of care for the lesbian, gay, bisexual, and transgender community. Dermatol Clin..

[3] https://bancos.salud.gob.ar [Internet] Atención de la salud integral de personas trans, travestis y no binarias. Guía para equipos de salud. 2º edición; 2020 [cited 2023 Oct 19]. Available from: https://bancos.salud.gob.ar/sites/default/files/2020-10/guia-salud-personas-trans-travestis-nobinarias.pdf ***.

[4] Boothby-Shoemaker W., Mansh M., Sternhell-Blackwell K., Peebles J.K. (2023). Sexual orientation and gender identity inclusion in dermatology research: a 10-year analysis. J Am Acad Dermatol.

[5] Shahwan K.T., Nosewicz J., Trinidad J., Carr D.R. (2022). Sexual and gender minority publication trends in the dermatology literature. Arch Dermatol Res..

[6] https://sad.org.ar/ [Internet] Guía para la atención respetuosa de la diversidad de género de las personas; 2022. [cited 2023 Oct 19]. Available from: https://sad.org.ar/wp-content/uploads/2022/12/Guia-para-una-atencion-respetuosa-de-la-diversidad-de-genero-de-las-personas-adaptada-a-la-consulta-dermatologica.docx.pdf ***.

[7] Motosko C.C., Tosti A. (2021). Dermatologic care of hair in transgender patients: a systematic review of literature. Dermatol Ther (Heidelb)..

[8] Ravindran S., Nazeer M., Criton S. (2023). Dermatologic care of the lesbian, gay, bisexual and transgender community of India. Indian J Dermatol Venereol Leprol..

[9] Becasen J.S., Denard C.L., Mullins M.M., Higa D.H., Sipe T.A. (2019). Estimating the prevalence of HIV and sexual behaviors among the US transgender population: a systematic review and meta-analysis, 2006–2017. Am J Public Health..

[10] Jia J.L., Polin D.J., Sarin K.Y. (2020). Ways to improve care for LGBT patients in dermatology clinics. Dermatol Clin..

[11] Thoreson N., Park J.A., Grasso C., Potter J., King D.S., Marc L.G. (2021). Incidence and factors associated with acne among transgender patients receiving masculinizing hormone therapy. JAMA Dermatol..

